# Changes in Ventilation Practices for Bronchiolitis in the Hospital Ward and Need for ICU Transfer over the Last Decade

**DOI:** 10.3390/jcm11061622

**Published:** 2022-03-15

**Authors:** Ruth Solana-Gracia, Vicent Modesto i Alapont, Leticia Bueso-Inchausti, María Luna-Arana, Ariadna Möller-Díez, Alberto Medina, Begoña Pérez-Moneo

**Affiliations:** 1 Department of Paediatrics, Hospital Universitario Infanta Leonor y Hospital Virgen de la Torre, 28031 Madrid, Spain; begona.perezm@salud.madrid.org; 2 Paediatric Intensive Care Unit, Hospital Universitario La Fe, 46026 Valencia, Spain; vicent.modesto@gmail.com; 3 Faculty of Medicine, Universidad Complutense de Madrid, 28040 Madrid, Spain; leticia.bueso@gmail.com (L.B.-I.); marlunaa02@gmail.com (M.L.-A.); amollerdiez@gmail.com (A.M.-D.); 4 Paediatric Intensive Care Unit, Hospital Universitario Central de Asturias, 33011 Oviedo, Spain; amedinavillanueva@gmail.com

**Keywords:** continuous positive airway pressure, viral bronchiolitis, non-invasive ventilation, oxygen inhalation therapy, respiratory insufficiency

## Abstract

There is limited evidence of the potential benefits of the use of high-flow nasal cannula (HFNC) for the management of bronchiolitis in the ward. Our aim is to describe the ventilation trends for bronchiolitis in our hospital along with the introduction of an HFNC ward protocol and to determine the need for respiratory support escalation and transfer to an intensive care unit (ICU). A retrospective analytical observational study of children < 12 months old requiring admission for a first RSV bronchiolitis episode in a single centre from January 2009 to December 2018. The sample was divided into four groups according to the type of respiratory support that would ensure the clinical stability of the infants on admission. A total of 502 infants were recruited. The total number and percentage of patients admitted in the ward grew progressively over time. Simultaneously, there was an increase in HFNC and, paradoxically, an increase in ICU transfers. The risk of failure was higher for those who required HFNC or CPAP for clinical stabilisation in the first 12 h after admission. Moreover, the risk of failure was also higher in children with standard oxygen therapy promptly escalated to HFNC, especially if they had atelectasis/viral pneumonia, coinfections or a history of prematurity. Despite the limitations of a retrospective analysis, our study reflects usual clinical practice and no correlation was found between the usage of HFNC and a shorter length of hospital stay or less time spent on oxygen therapy.

## 1. Introduction

The annual incidence of respiratory syncytial virus (RSV) bronchiolitis is 16.4% for children < 2 years of age in Spain. Around 3% of children of this age are admitted to hospital, bronchiolitis being the main reason for admission [1]. However, therapeutic support measures (maintaining a correct state of hydration and nutrition) and the application of adequate respiratory support are still key for this clinical condition [2].

In recent years, the ease of application and safety of the high-flow nasal cannula (HFNC) has spread [3]. It was initially exclusive to intensive care units (ICUs) but now is present in conventional wards [4]. The clinical results of this change are highly controversial [5,6].

Determining the appropriate moment for ICU transfer is especially relevant for secondary-level hospitals to minimise time delays, appropriately mobilise resources and maintain patients in their best clinical condition. Therefore, our main goal was to analyse whether early HFNC to support the first RSV bronchiolitis in otherwise healthy inpatients < 12 months age was associated with a decrease in the need for respiratory support escalation and percentage of ICU referrals. Secondary objectives were the analysis of possible predictive factors for both outcomes and the description of the epidemic evolution and treatment of this condition in our hospital.

## 2. Materials and Methods

This is a retrospective analytical observational study of historical cohorts. The setting is a single secondary-level hospital facility. The recruitment period was uninterrupted from January 2009 to December 2018. The sampling was consecutive, enrolling those patients < 12 months old with a first RSV bronchiolitis episode admitted to the paediatric ward. RSV detection was determined by nasal washing antigen test. Infants with chronic pathologies (congenital heart disease, chronic lung disease, upper airway obstruction, craniofacial malformations, chromosomal diseases, syndromes) and those with negative RSV tests were excluded in order to improve the homogeneity of the sample and avoid the influence of epidemiological changes of other etiological agents.

HFNC delivery systems were: Optiflow Junior RT330 system and AIRVOTM2 (Fisher & Paykel Healthcare). From July 2012, a total of five devices have been available in our hospital. Its use depended on the prescribing physician’s criteria as a therapeutic escalation to treat hypoxemia and/or work of breathing after failure of standard oxygen therapy (SOT). General targets were to keep oxygen saturation ≥ 92% with a respiratory and heart rate within the normal range for age [7]. HFNC could be started at the emergency room or at any time during the evolution in the ward, and the initial flow was 1.5–2 L/kg/min. Other demographic and clinical data were also collected as covariates: age, sex, duration of respiratory support (any type), length of hospital stay, duration of ICU admission, use of other treatments (fluid therapy, adrenaline, bronchodilators, corticosteroids or antibiotics). In those patients < 3 months old (<6 kg), the use of continuous positive airway pressure (CPAP) or bilevel positive airway pressure (BLPAP) was also documented.

The main outcome variable was failure of treatment, defined as a need for ICU transfer or escalation to another modality of respiratory support.

The sample was divided into four groups according to the type of respiratory support that would ensure the clinical stability of the infants for at least 12 h after admission:

- Non-support group—admitted without respiratory support and stable for at least 12 h. Failure means to be started on SOT, HFNC, CPAP/BLPAP or ICU transfer.
- SOT group—stability reached on SOT. Failure means escalation of respiratory support (HFNC/CPAP/BLPAP) or ICU transfer.
- HFNC group—stability achieved on HFNC. Failure means that CPAP/BLPAP is subsequently required or ICU transfer.
- CDP cohort—initial stabilisation with a continuous distension pressure (CDP) device in the airway (CPAP and/or BLPAP). Failure means the need for ICU transfer.

Likewise, the following complications were defined: atelectasis, pneumothorax, viral pneumonia and death. In addition, the following coinfections were also recorded: Bordetella pertussis, bacterial pneumonia, acute otitis media, urinary tract infection and sepsis. Coinfections and complications did not mean exclusive categories.

The data were obtained through the digital medical records using activity analysis by main diagnosis (ICD-9 codes until March 2017 and later ICD-10 codes of RSV bronchiolitis and RSV pneumonia) in an anonymised way, guaranteeing confidentiality based on Organic Law 3/2018 on Protection of Personal Data and Guarantee of Digital Rights. The study protocol was approved by the Hospital’s Research Ethics Committee (Ref 07/616954.9/21 approval with the omission of request for informed consent).

For data analysis, the statistical package Stata 13.1 (College Station, TX, USA) and the free software statistical package R 4.0.3 were used. Description of the categorical variables was made using percentages. For continuous variables, if they were normal (Kolmogorov–Smirnov and/or Shapiro–Wilks) as mean and standard deviation (SD) and if not, with medians and interquartile ranges. Bivariate analyses of categorical variables were performed with the Chi-square test or Fisher’s exact test; continuous variables were analysed with the Student’s *t*-test if they were normal or with their non-parametric equivalents if otherwise. The description of the survival time variables was made using the Kaplan–Meier graphical method, and the log-rank test was used for bivariate comparison. In the case of survival times in competitive temporal event variables, the cumulative incidence representation for competitive risks was used.

For multivariate analyses, regression models were fitted using a general linear model and a Cox proportional hazards model for survival times. In order to gain statistical power, for this analysis, we put together complications and coinfections in one single predictor variable but excluding acute otitis media because there is no biological plausibility for its impact on the need for respiratory support. Selection of candidate variables in the multivariable regression was based on comparisons of information entropy measures of predictive accuracy of the model, using Akaike’s information criterion (AIC). The Fine–Gray model was used for the multivariate study of competing risk [8]. To estimate the causal effects of the kind of respiratory support on the probability of ICU admission, the logistic regression (LR) models were adjusted using propensity scores [9]. For the computation of the c-statistic, we used the DeLong method. For all comparisons, a result was considered statistically significant when the *p*-value was <0.05.

As this is a retrospective study, we performed a power calculation using the actual study sample size. There is approximately a 12% probability that a patient with bronchiolitis requires ICU transfer when treated in a secondary-level hospital [10]. Assuming a *p* = 0.05 probability (alpha) of type I error and considering a clinically relevant reduction of up to 7% in ICU transfer (near halving the proportion, but only a five percentage point difference), a cohorts study with a sample size of 70 HFNC patients and 362 SOT patients can reject the null hypothesis of equal probability of ICU transfer with a power estimation of 24.7%. The calculated probability (beta) of making a type II error (to affirm the null hypothesis as true) is 75.3%.

## 3. Results

The number of recruited infants was 502. Description of the demographic variables, treatment and clinical evolution of all infants for the total sample and depending on the modality of respiratory support required for initial stabilisation is detailed in Table 1. There were no cases of pneumothorax or deaths. The ratio of ICU transfer was clearly higher for HFNC and CDP groups (*p* < 0.001). Patients who did not receive any initial respiratory support were not subsidiaries of ICU transfer.

**Figure 1:**
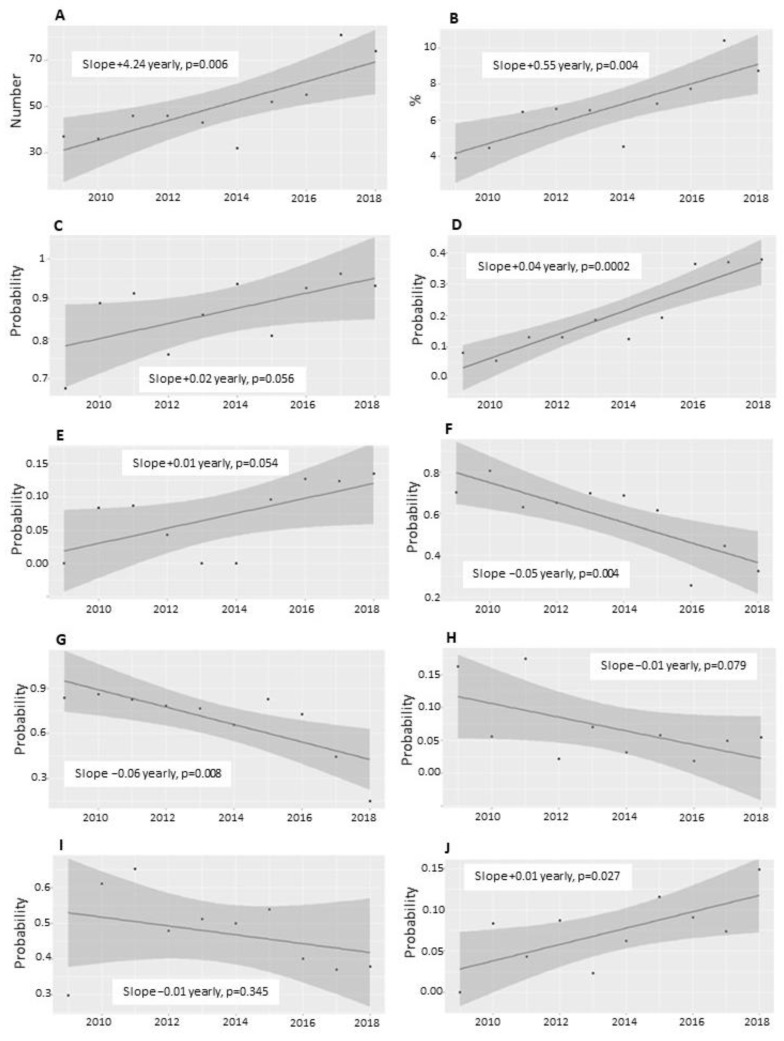
Incidence of RSV bronchiolitis admission, respiratory support therapies, medical treatment and evolution per year. The trend in ICU transfer in our hospital has been upward since 2009, coinciding with an increase in the number of admissions for bronchiolitis. The odds ratio (OR) of ICU transfer per year is 1.15 (95% CI: 0.991–1.32; *p* = 0.07). (**A**): Number of annual admissions for RSV bronchiolitis in the ward. (**B**): Percentage of annual admissions due to RSV bronchiolitis in the ward. (**C**): Probability of using some type of oxygen therapy. (**D**): Probability of using HFNC. (**E**): Probability of using CPAP/BLPAP. (**F**): Probability of using beta2-mimetics. (**G**): Probability of using adrenaline. (**H**): Probability of using steroids. (**I**): Probability of using fluid therapy. (**J**): Probability of ICU transfer.

The cohort evolution in the different epidemic periods is reflected in Figure 1. The total count and percentage of discharged patients with RSV bronchiolitis related to the total number of discharges (any cause) per year increased progressively (0.5% annually, *p* < 0.01), being especially high from 2017 onwards. There was an annual 3.7% increase in HFNC use (*p* < 0.001) and a 1% increase in ICU transfers (*p* = 0.03). The graphs also show a statistically significant upward trend in the use of any type of oxygen therapy and CPAP/BLPAP. During the period of the study, inhaled betamimetics and epinephrine were used significantly less (from 90% of patients in 2009 to 30–40% in 2018). The decrease in the use of oral corticosteroids was less pronounced. Differences over time for antibiotics (≈23%) or intravenous fluid therapy (45–55%) could not be found. The probability of invasive mechanical ventilation was very low and did not vary significantly over time: year 2009: 0/38 (0%); year 2010: 2/36 (5.6%); year 2011: 0/46 (0%); year 2012: 0/46 (0%); year 2013: 0/43(0%); year 2014: 1/32 (3.1%); year 2015: 2/52 (3.8%); year 2016: 1/55 (1.8%); year 2017: 0/81 (0%); year 2018: 2/74 (2.7%) (*p* = 0.2209; Fisher’s exact test for count data) (none in 2009, 2011, 2012, 2013 or 2017; *n* = 1 in 2014, 2016; *n* = 2 in 2010, 2015 and 2018).

**Figure 2:**
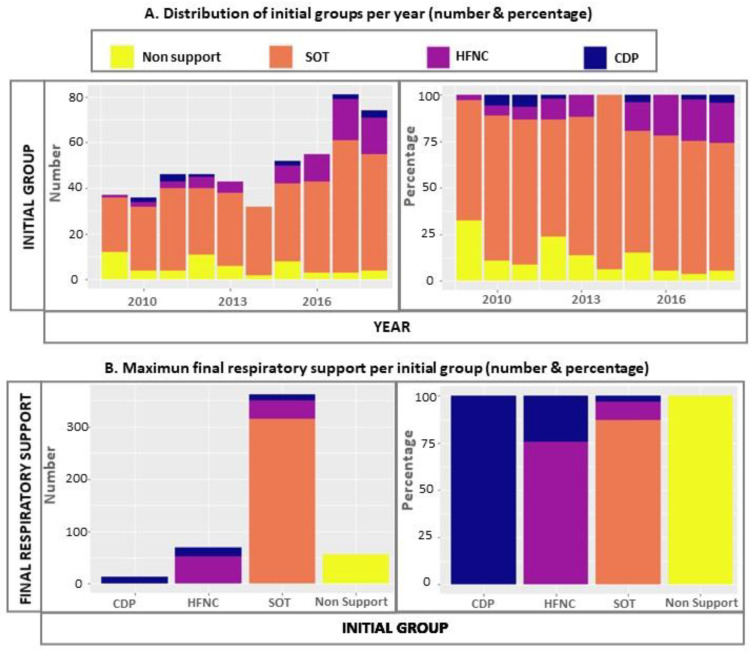

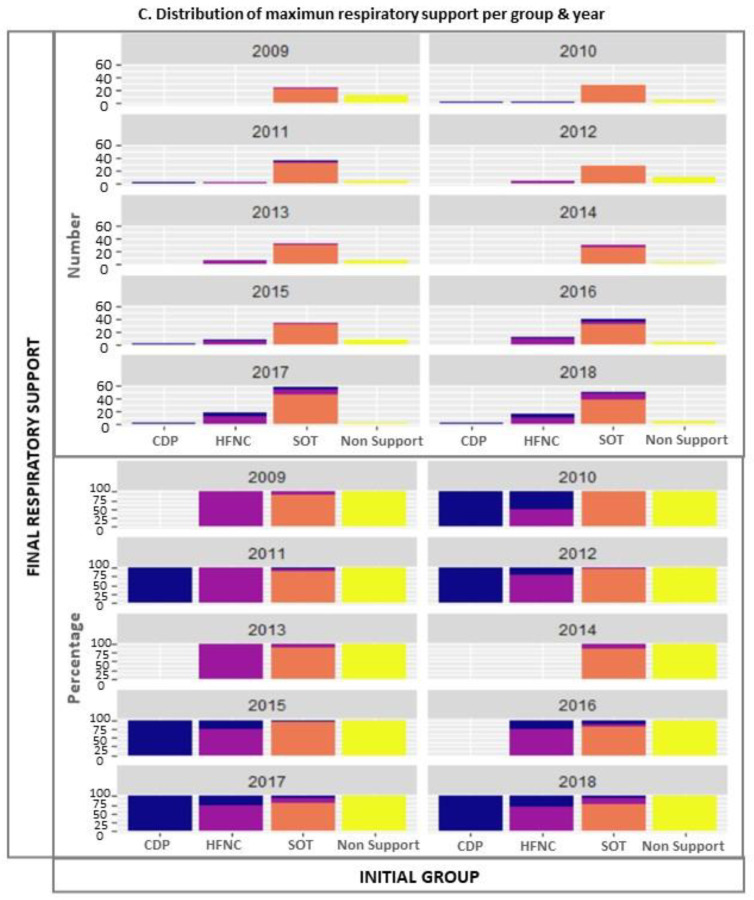
Distribution of initial groups per year and its maximum escalation of respiratory support in the hospital ward: (**A**) Total distribution of the groups according to the respiratory modality required for initial stabilisation; (**B**) maximum respiratory support for each of these groups during admission; (**C**) maximum escalation of respiratory support for each of these groups per year.

The global and per year distribution of the groups on admission and their maximum respiratory support escalation is represented in Figure 2. The most commonly available and more frequently used modality of respiratory support was SOT. There were no cases of respiratory modality support escalation for stabilised patients without the need for oxygen. 

The maximum respiratory support for the patients transferred to ICU was: invasive mechanical ventilation 20.5% (8/39), BLPAP 23.1% (9/39), CPAP 30.8% (11/39) and HFNC 30.8% (11/39).

ICU transfer or therapeutic escalation predictors, using logistic regression models, for the whole sample and each group are represented in Table 2.

Regarding ICU transfer, this multivariate analysis (adjusted by epidemic season) confirmed a statistically higher risk for HFNC and CDP groups compared with the SOT group. Another important factor was the presence of complications (atelectasis) and coinfections. For the SOT group, in addition to complications, the main factor associated with ICU transfer was escalation to HFNC (OR 29.1, 95% CI: 5.6–150; *p* < 0.001). For the HFNC group, the duration (days) on HFNC behaved as a protective factor (OR 0.48; 95% CI: 0.3–0.78; *p* = 0.002). There were no statistically significant results for the CDP group.

The risk of respiratory therapy escalation (Table 2) varied depending on the epidemic season (OR 1.26, 95% CI: 1.13–1.40; *p* < 0.001). In Figure 2C, an upward trend for respiratory support escalation can be observed from 2016 onwards. Prematurity, complications/coinfections, HFNC and CPD groups were also global risk factors. Within the SOT group, in addition to prematurity and complications/coinfections, this upward trend over the years was statistically significant (OR 1.43, 95% CI: 1.22–1.68; *p* < 0.001) and the duration (days) on SOT behaved as protector (OR 0.31, 95% CI: 0.20–0.48; *p* < 0.001). Within the HFNC cohort, as well as for ICU transfer, the duration (days) on HFNC was a protective factor (OR 0.49, 95% CI: 0.32–0.75; *p* < 0.001). For the CDP cohort, we did not find any statistically significant predictor.

**Figure 3:**
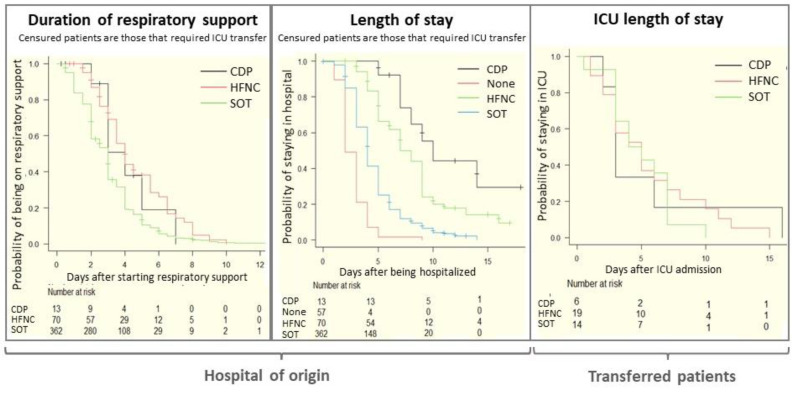
Kaplan–Meier graphics by groups for duration of respiratory support and hospital length of stay in our hospital (hospital of origin) and ICU stay for those who were transferred.

In survival studies (Figure 3), censoring patients transferred to the ICU, the total duration of oxygen therapy was slightly less for the SOT group than the HFNC and CDP groups. The longest length of total hospital stay was for the CDP group, followed in order by the HFNC, SOT and non-support groups. There were no differences among groups in length of ICU stay.

**Figure 4:**
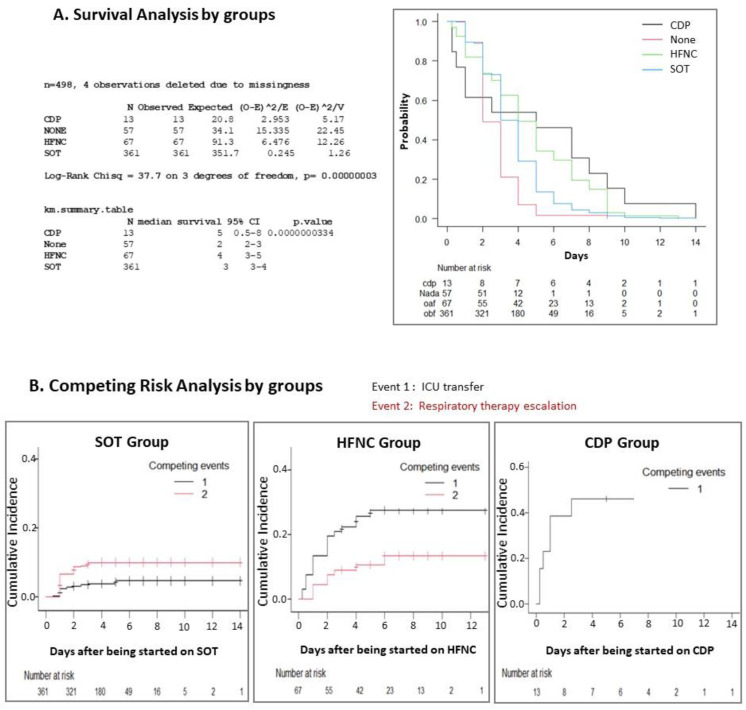
Survival and competing risks analysis by groups. (**A**) Survival analysis: time free from respiratory therapy escalation or ICU transfer by groups. (**B**) Competing risk analysis to determine which event (1. ICU transfer, 2. Respiratory therapy escalation) occurred first by groups: Event 1, ICU transfer; Event 2, respiratory therapy escalation.

Using a competing risk analysis (Events = ICU transfer and respiratory support therapy escalation), patients who remained in the same initial modality of respiratory support (same group) until discharge were censored (Figure 4). The unsupported group had no ICU transfer or escalation. For the SOT group, the cumulative incidence of therapeutic escalation was higher than ICU transfer, in contrast to what happened in the HFNC group. In the Fine–Gray multivariate study, adjusting by epidemic season, the only statistically significant variable related to ICU transfer was the initial group classification (HR = 4.375, 95% CI: 3.006–6.368; *p* < 0.0001).

Finally, to estimate the specific causal effect of HFNC use, three logistic regression models were adjusted with control for confusion (year of epidemic season and complications) using a propensity score. In the first, selecting data only from the SOT and HFNC groups, the use of HFNC behaved as a risk factor for ICU transfer when compared with SOT (ORa = 1.22, 95% CI: 1.1–1.34, *p* = 0.000105; c-statistic = 0.719, 95% CI: 0.636–0.803). In the second, selecting data only from SOT and CDP groups, the use of CPAP/BLPAP behaved as a risk factor for ICU admission when compared with SOT (ORa = 1.04, 95% CI: 1.01–1.07, *p* = 0.014; c-statistic = 0.634, 95% CI: 0.54–0.74). As a result, the magnitude of this effect was less than the previous comparison with HFNC. In the third, selecting data only from the HFNC and CDP cohorts, the use of CPAP/BLPAP did not have a significant influence on ICU admission when compared with HFNC (ORa = 0.701, 95% CI: 0.258–1.9, *p* = 0.487; c-statistic = 0.513, 95% CI: 0.357–0.668).

## 4. Discussion

The ratio and total count of admissions due to RSV bronchiolitis have increased over the last decade, especially from 2017 onwards, representing as much as 10% out of all admissions.

Respiratory therapies have been more frequently used over time to achieve clinical stability in the ward (Figure 1), and SOT has been the most commonly used (up to 72% of admissions). According to guidelines [2], the administration of inhaled betamimetics and adrenaline has gone from universal use in all patients in 2009 to only one-third of patients recently. Betamimetics were most frequently used for patients without respiratory support or with SOT. On the contrary, fluid therapy and antibiotics stood out for HFNC and CPD groups, probably related to more severe conditions and coinfections (especially bacterial pneumonia, Table 1).

Overall, 25% of the patients had some type of complication/coinfections and 7.8% of the patients required ICU transfer. Those with the highest percentage of transfers was CDP (46%), followed by HFNC group (27%).

After five HFNC devices were routinely available in our hospital in 2012, the increase in their usage in the ward has been evident, particularly since 2016 (Figure 1). Globally, the probability of respiratory escalation has been 17%, but there has also been an upward trend (Figure 2C), 1.26 OR, for each epidemic season (Table 2). This, paradoxically, has been related to a higher probability of ICU transfer. The patients who have been intubated have been very scarce (*n* = 8) and without significant trends over time.

Analysing the results on the basis of the type of respiratory support necessary for their initial stabilisation, the percentage of respiratory escalation and ICU transfer was clearly higher for the HFNC group (41% and 27%, respectively) and CPD group (46% in both situations) than the other groups (Table 1). The multivariate analysis for prediction of therapeutic failure (escalation or ICU transfer), in general, reinforces this finding and points out as other associated factors the presence of atelectasis/pneumonia and a history of prematurity. In the analysis between groups, the permanence in the same respiratory support modality for the patients with SOT or HFNC during the first 3–4 days of admission was related to a lower probability of therapeutic failure (Table 2 and Figure 3). We understand this as a logical finding since the natural evolution in such cases leads to improvement. In SOT patients, the odds ratio (OR) of ICU transfer for those who required escalation to HFNC was 29.1 (95% CI: 5.6–150; *p* < 0.0001) compared with those who did not need it. Adjusting for the effect of the epidemic season and the presence of complications, HFNC and CDP patients maintained significantly higher odds of ICU transfer compared with SOT patients.

In the analysis of competitive risks for the HFNC group, the higher incidence of ICU transfer compared with therapeutic escalation is conditioned by our limited availability of CDP devices just for patients < 3 months old (<6 kg).

Finally, the length of hospital stay was longer in children who required some type of respiratory support compared with those non-supported or with SOT. A shorter length of hospitalisation for the HFNC group could not be found. The total duration of oxygen therapy was slightly less for the SOT group than the HFNC or CDP groups. There were no differences in length of ICU stay among groups.

Unlike what happened in our hospital, the incidence of bronchiolitis hospitalisations among US children from 2000 to 2016 declined [11]. However, as in our study, the proportion of bronchiolitis hospitalisations among overall hospitalisations increased from 16% to 18%. There was a progressive increase in non-invasive mechanical ventilation use (especially from 2012 onwards), and the nationwide hospital direct cost became greater. The overall survival rate was 99.95%. Moreover, the percentage of bronchiolitis admissions transferred to ICU grew from 11.7% in 2010 to 24.5% in 2019 [12], and the application of mechanical ventilation kept constant over time (≈3%). The paradoxical tendency to increase ICU utilisation after the adoption of a ward-based protocol had already been described [13]. Similar results have been found in Canadian paediatric centres, as the initiation of HFNC resulted in no significant change in intubation rates or hospital length of stay but had an increase in ICU admissions, concluding that the overall disease course is not modified by the use of HFNC [14].

Previous randomised control trials suggested that CPAP may be more efficient than HFNC for initial respiratory support in young infants hospitalised in ICU for moderate to severe acute viral bronchiolitis [15] and that the HFNC flow rate of 3 L/kg/min did not reduce the risk of therapeutic failure versus 2 L/kg/min [16]. Another open randomised trial found similar effects of respiratory rate, pCO_2_ or modified Woods Clinical Asthma Score comparing HFNC with binasal prong CPAP, treatment failure was scarce and no significant differences in treatment duration or length of stay were observed [17].

After verifying its ease of use and suspecting a possible greater comfort for the patient, the expansion of HFNC in hospital wards has been notable, but there has been limited evidence about its effects and safety. In this setting, when HFNC or SOT are randomly assigned, an initial clinical trial showed a lower rate of escalation of care due to treatment failure when HFNC was used early during the hospital admission than when SOT was used [18]. The percentage of infants receiving escalation of care was 12% in the HFNC group compared with 23% in the SOT group (*p* < 0.001), and the rate of ICU transfer was similar (12% v. 9%, respectively; *p* = 0.08). We presume that these results from previous studies are not comparable with ours, given that the criteria for initiating HFNC in the former studies were not based on the clinical condition, and possibly many of the children who received HFNC did not need it. In fact, the cost-effectiveness of this approach would be in doubt [19,20]. Moreover, the statement of this possible beneficial effect of HFNC has been contradictory with that expressed by a recently published clinical trial [21]. In addition, no differences in duration of hospital stay, ICU stay or oxygen therapy could be found [18], similar to our results. Surprisingly, at the time of therapeutic escalation, patients receiving HFNC had higher respiratory rates than those receiving SOT (62 v. 54, respectively; *p* < 0.001) [18]. This could have happened, perhaps, because of the clinician’s over-reliance on the HFNC itself. Another trial suggested that, if used as a rescue therapy for children who are not adequately supported by SOT, HFNC might reduce the proportion of ICU admissions, but the bed-day costs were equivalent in both groups and consumables cost of HFNC was 16 times that of SOT [22].

Different systematic reviews/meta-analyses have recently been published in search of evidence of the HFNC effects and benefits on bronchiolitis [23,24,25,26,27]). Lin et al. [23] concluded that HFNC is safe as initial respiratory management, but it did not significantly benefit children with bronchiolitis compared with SOT and nasal CPAP. HFNC may decrease the rate of treatment failure for children with bronchiolitis compared with SOT [23,24]. When comparing CPAP v. HFNC, in moderate/severe bronchiolitis, CPAP demonstrated a lower risk of therapeutic failure (especially when using a helmet interface) and a longer time to failure, but more adverse events such as nasal injury [25,26]. However, the use of CPAP in the hospital ward is even more controversial. The increasing prevalence of HFNC as therapy for bronchiolitis without clear evidence has also led to research into how to avoid overuse [28].

The main weakness of our study is that it is a retrospective analysis of the evolution and results of real clinical practice in a secondary-level hospital. As there is no randomisation, it is not possible to establish the causal responsibility of each ventilation mode and our inferences always are, even with causal methods of analysis like propensity scoring matching, submitted to residual confounding. With samples not large enough to contain sufficient outcomes, the number of covariates that can be included in the models and residual confounding can be problematic [29]. Another source of concern is that our results could not be adjusted to bronchiolitis severity scales, as they were not systematically collected in the clinical records. The recruitment of children < 1-year-old may yield worse clinical outcomes than if the sample had been extended to <2-year-olds since younger age is a negative prognostic factor, but most of the patients admitted to our hospital with a first episode of this condition are this age.

In light of the results and despite the limitations, our study reflects the usual clinical practice of the unit. The initial clinical evaluation and stabilisation in the first 12 h after admission made by physicians seems to be adequate since the unsupported patients did not require escalation or ICU transfer. Moreover, the higher risk of these events occurred in the HFNC group, CDP group and in those of the SOT group who were rapidly escalated to HFNC.

## 5. Conclusions

The number and percentage of patients < 1 year admitted for RSV bronchiolitis among all-cause admissions has grown progressively in the last 10 years. The availability of HFNC and the introduction of a ward protocol for patients with this pathology caused an increase in their use over time, and, paradoxically, an increase in ICU transfers was also observed. The children most at risk of therapeutic escalation or ICU transfer were those who required HFNC or CPAP for clinical stabilisation in the first 12 h after admission, especially if they had atelectasis, viral pneumonia and coinfections or a history of prematurity. In addition, children with SOT who required escalation with HFNC during the following 2–3 days after admission also had a greater risk of ICU transfer. No differences were found in length of ICU stay among groups, and there was no correlation between the usage of HFNC and a shorter length of hospital stay or total duration of oxygen therapies. In the future, it would be of great interest to carry out more studies on the effect of HFNC in patients who have failed with SOT.

## Figures and Tables

**Table 1:** Description of the sample: global and by initial respiratory support groups.

Variables	Total	Non-Support Group	SOT Group	HFNC Group	CDP Group	*p*-Value
***n* (%)**	502	57 (11.3)	362 (72.1)	70 (13.9)	13 (2.6)	
**Demographic variables**						
**Male (%)**	274 (54.6)	37 (64.9)	196 (54.1)	36 (51.4)	5 (38.5)	0.25
**Age (months)** **(median [IQR])**	3 [2; 5]	3 [2; 4]	3 [2; 4]	2.5 [2; 4]	1 [1; 2]	0.0012
<1 months (%)	60 (12)	8 (14.0)	33 (9.1)	11 (15.7)	9 (69.2)	
1–3 months (%)	236 (47)	32 (56.1)	167 (46.1)	34 (48.6)	3 (23.1)	
3–6 months (%)	134 (26.7)	11 (19.3)	108 (29.8)	14 (20.0)	1 (7.7)	
>6 months (%)	72 (14.3)	6 (10.5)	54 (14.9)	11 (15.7)	0	
**Prematurity < 37 weeks (%)**	42 (8.4)	3 (5.3)	31 (8.6)	7 (10.0)	1 (7.7)	0.80
**Gestational age (weeks) if prematurity (median [IQR])**	34.5 (33; 36)	36 (33; 36)	34 (33; 35)	33 (28; 36)	35	0.71
**Medical treatment**						
**Bronchodilators (%)**	418 (83.3)	47 (82.5)	312 (86.2)	52 (74.3)	7 (53.9)	0.004
Beta2-mimetics (%)	272 (54.2)	28 (49.1)	213 (58.8)	27 (38.6)	4 (30.8)	0.004
Adrenaline (%)	320 (63.7)	40 (70.2)	233 (64.4)	41 (58.6)	6 (46.2)	0.30
**Corticoids (%)**	33 (6.6)	0	31 (8.6)	1 (1.4)	1 (7.7)	0.10
**Antibiotics (%)**	113 (22.5)	10 (17.4)	70 (19.3)	25 (35.7)	8 (61.5)	<0.0001
**Fluid therapy (%)**	231 (45.6)	17 (29.8)	142 (39.2)	60 (85.7)	12 (92.3)	<0.0001
**Clinical evolution**						
**Complications (%)**	126 (25.1)	12 (21.1)	78 (21.6)	27 (38.6)	9 (69.2)	<0.0001
Atelectasis (%)	33 (6.6)	0 (0)	15 (4.1)	15 (21.4)	3 (23.1)	<0.0001
Viral pneumonia (%)	6 (1.2)	1 (1.8)	3 (0.8)	2 (2.9)	0	0.30
Pneumothorax (%)	0 (0)	0 (0)	0 (0)	0 (0)	0 (0)	0 (0)
Death (%)	0 (0)	0 (0)	0 (0)	0 (0)	0 (0)	0 (0)
**Coinfections (%)**	124 (24.7)	11 (19.3)	77 (21.3)	27 (38.6)	9 (69.2)	<0.0001
*Bordetella pertussis* (%)	6 (1.2)	1 (1.8)	4 (1.1)	0	1 (7.7)	0.15
Bacterial pneumonia (%)	44 (8.8)	3 (5.3)	25 (6.9)	11 (15.7)	5 (38.5)	0.001
Acute otitis media (%)	35 (6.7)	4 (7.0)	31 (8.6)	0	0	0.03
Urinary tract infection (%)	4 (0.8)	1 (1.8)	3 (0.8)	0	0	0.52
Sepsis (%)	2 (0.4)	1 (1.8)	0	0	1 (7.7)	0.12
**Total duration of respiratory support (days) (median [IC95%])**	3 (3–3)	0 (0)	3 (2.5–3)	4 (3.5–5.5)	4 (2-NA *)	<0.0001
**Hospital length of stay (days) (median [IC95%])**	4 (4–4)	2 (2–3)	4 (4–4)	8 (6–9)	10 (7-NA)	<0.0001
**Failure of initial respiratory support (%)**	84 (16.6)	0 (0)	49 (13.5)	29 (41.4)	6 (46.2)	<0.0001
**ICU transfer (%)**	39 (7.8)	0 (0)	14 (3.9)	19 (27.1)	6 (46.2)	<0.0001
Invasive mechanical ventilation (% Total) (% ICU transferred)	8 (1.5) (20.5)	NA	3 (0.8) (21.4)	3 (4.2) (15.8)	2 (15.3) (33.3)	0.004
ICU length of stay (days) (median [IC95%])	4 (3–6)	NA	4.5 (3–7)	5 (3–7)	3 (2-NA)	0.883

* NA: Not available.

**Table 2:** Predictive multivariate LR model for ICU transfer and respiratory support escalation.

Independent Variables	Odds Ratio	95% Confidence Interval		*p* Value
		Lower Limit	Upper Limit	
**PREDICTOR FACTORS FOR ICU TRANSFER**				
**GLOBAL SAMPLE**				
Intercept	2.7 × 10^−127^	1.59 × 10^−254^	4.57	0.051
Epidemic season	0.14	0.07	1.93	0.056
Complications/Co-infections	6.69	3.04	14.7	<0.001
Prematurity	2.57	0.85	7.77	0.09
Cohorts:				
• SOT (Ref)	-	-	-	-
• No support	2.95 × 10^−7^	0	Inf	0.98
• HFNC	5.86	2.61	13.2	<0.001
• CDP	10.6	2.84	39.6	<0.001
**SOT GROUP**				
Intercept	0.004	0.001	0.021	<0.001
Complications/Co-infections	3.92	1.06	14.5	0.04
Escalation to:				
• HFNC	29.1	5.63	150	<0.001
• CDP	3.03	0.62	14.7	0.17
**HFNC GROUP**				
Intercept	1.22	0.32	4.66	0.77
Complications/Co-infections	5.11	1.38	18.9	0.01
Days on HFNC	0.48	0.3	0.78	0.002
**PREDICTOR FACTORS FOR RESPIRATORY SUPPORT ESCALATION**				
**GLOBAL SAMPLE**				
Intercept	2.42 × 10^−203^	9.55 × 10^−298^	6.15 × 10^−109^	<0.001
Epidemic season	1.26	1.13	1.4	<0.001
Complications/Co-infections	4.72	2.56	8.69	<0.001
Prematurity	3.04	1.35	6.85	0.007
Cohorts:				
• SOT (Ref)	-	-	-	-
• No support	7.95 × 10^−8^	0	Inf	0.98
• HFNC	2.71	1.46	5.05	0.001
• CDP	3.16	0.93	1.07	0.07
**SOT GROUP**				
Intercept	4.13 × 10^−313^	0	3.61 × 10^−173^	1.21 × 10^−5^
Epidemic season	1.43	1.22	1.68	<0.001
Prematurity	3.72	1.25	11.1	0.02
Complications/Co-infections	9.23	3.74	22.8	<0.001
Bronchodilators	2.54	0.9	7.14	0.07
Days on O_2_	0.31	0.2	0.48	<0.001
**HFNC GROUP**				
Intercept	5.17	1.08	24.7	0.04
Complications/Co-infections	5.27	1.42	19.6	0.01
Bronchodilators	0.37	0.09	1.47	0.16
Days on HFNC	0.49	0.32	0.75	<0.001

## Data Availability

Clinical data files are stored at Infanta Leonor University Hospital. It may be shared if needed.
